# Evidence for fomite transmission of SARS‐CoV‐2 Omicron variant in a mouse model

**DOI:** 10.1002/mlf2.70022

**Published:** 2025-06-24

**Authors:** Sidi Yang, Liu Cao, Kun Li, Tiefeng Xu, Zixiao Yang, Yanxi Ji, Lihong Liu, Birong Zheng, Changwen Ke, Xiaofang Peng, Hong Peng, Deyin Guo, Chun‐Mei Li

**Affiliations:** ^1^ Guangzhou National Laboratory Guangzhou International Bio‐Island Guangzhou China; ^2^ Centre for Infection and Immunity (CII), School of Medicine Shenzhen Campus of Sun Yat‐sen University Shenzhen China; ^3^ Institute of Human Virology, Department of Pathogen Biology and Biosecurity, and Key Laboratory of Tropical Disease Control of Ministry of Education, Zhongshan School of Medicine Sun Yat‐sen University Guangzhou China; ^4^ Center for Disease Control and Prevention of Guangdong Province Guangzhou China; ^5^ Department of Infectious Diseases the Third Affiliated Hospital of Sun Yat‐Sen University Guangzhou China; ^6^ State Key Laboratory of Respiratory Disease, National Clinical Research Center for Respiratory Disease Guangzhou Institute of Respiratory Health, the First Affiliated Hospital of Guangzhou Medical University Guangzhou China

## Abstract

Throughout the COVID‐19 pandemic, the risk of fomite‐based transmission of severe acute respiratory syndrome coronavirus 2 (SARS‐CoV‐2) has not been systematically investigated. In this study, we employed the K18‐hACE2 mouse infection model to experimentally assess the relative contribution of fomite transmission. Our findings indicate that while fomite transmission can occur in certain cases, the risk of fomite transmission in natural settings may be relatively low when appropriate hygiene practices are followed. These results may help optimize public health measures for more effective control of the COVID‐19 pandemic.

Severe acute respiratory syndrome coronavirus 2 (SARS‐CoV‐2), the causative agent of the global pandemic of coronavirus disease 2019 (COVID‐19), has emerged as a significant global public health threat. The latest addition to the list of variants of concern, Omicron, has been demonstrated to possess a higher basic reproduction number and potentially increased transmission compared to the Delta variant and the ancestral SARS‐CoV‐2 virus, thereby exerting significant pressure on epidemic prevention efforts^1^. There is overwhelming evidence that inhalation of SARS‐CoV‐2 is a primary route of transmission for widespread outbreaks^2^. However, the potential indirect transmission routes of SARS‐CoV‐2 and the relative contributions of different transmission routes, as well as the resulting infections, remain subjects of debate.

Fomite‐borne indirect transmission was thought to play a more prominent role at the beginning of the SARS‐Cov‐2 pandemic^3^, ^4^. Indeed, numerous studies have revealed that SARS‐CoV‐2 could persist on the fomites surfaces^5^, ^6^, ^7^. While some researchers believe that the risk of fomite transmission can be negligible^8^, ^9^, ^10^, ^11^, an increasing number of studies have suggested that fomite transmission cannot be discounted^12^, ^13^, ^14^, ^15^. Several studies have reported the detection of SARS‐CoV‐2 viral RNA on fomite surfaces, including those in various hospital indoor environments and cold chain foods. Nevertheless, only a few of studies could recover infectious SARS‐CoV‐2 from these fomites in virus culture^14^, ^16^, ^17^, ^18^. This limitation hampers our understanding of fomite transmission and its relative contribution. Thus, the examination of fomite transmission in pertinent experimental models serves as a valuable complement to epidemiological studies. The routes of fomite transmission for SARS‐CoV‐2, particularly the Omicron variant, have not been confirmed through laboratory documentation.

In this study, we utilized the K18‐hACE2 mouse infection model to experimentally explore the relative contribution of fomite transmission. To investigate viral contamination of ambient surfaces by infected animals, six K18‐hACE2 mice were intranasally inoculated with 1 × 10^5^ TCID_50_ of the SARS‐CoV‐2 Omicron variant and housed together in a single cage containing four types of inanimate surfaces: glass, stainless steel (SS), paper, and polystyrene. Four samples were collected from each inanimate surface every 2 days for up to 5 days post infection (dpi) (Figure 1A). Viral RNA was detectable in all samples at 1 dpi. At 3 dpi, viral RNA was detectable in 3 out of 4 (75%) glass samples, 2 out of 4 (50%) SS or paper samples, and all 4 out of 4 (100%) polystyrene samples. The detection of viral RNA at 5 dpi was similar to that at 3 dpi, but the average cycle threshold (CT) value of samples collected at 5 dpi (excluding SS) was lower than that observed at 3 dpi. Additionally, swabs were taken from surfaces within the cages to detect ambient contamination. The results indicated a similar trend of viral RNA detection on both ambient and inanimate surfaces. Viral RNA remained detectable until 5 dpi, with peak concentrations observed at 1 dpi, except for polystyrene, suggesting that K18‐hACE2 mice infected with SARS‐CoV‐2 can apparently contaminate the surrounding surfaces (Figure 1B).

**Figure 1:**
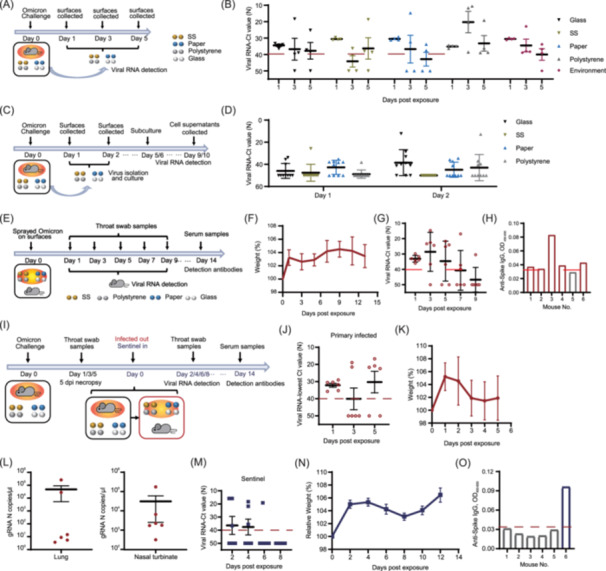
Laboratory evidence of SARS‐CoV‐2 fomite transmission. (A) A graphic outline of the experimental design and sample collection, detailing the detection of contaminated viral RNA from the ambience of infected K18‐hACE2 mice (*n* = 6). (B) Viral genomic RNA (gRNA) detected on inanimate surfaces, including glass, stainless steel (SS), paper, polystyrene, and the inner surface of cages, by quantitative reverse transcription‐polymerase chain reaction (qRT‐PCR) analysis targeting the nucleocapsid (N) gene. (C) A schematic diagram illustrating the isolation of viable virus from inanimate surfaces. (D) Viral gRNA measured in cell supernatants by qRT‐PCR targeting the N gene. Inanimate surfaces were collected for the indicated time points. Vero cells were used to culture virus from any contaminated inanimate surfaces. The cell supernatants were collected and viral gRNA was measured by RT‐PCR analysis of N. (E) A graphic outline of the experimental design and sample collection illustrating fomite transmission in K18‐hACE2 mice (*n* = 6). (F) The changes in body weight in the K18‐hACE2 mice (*n* = 6). (G) SARS‐CoV‐2 viral RNA load measured from throat swab at the indicated time points by qRT‐PCR analysis of N. (H) Reactivity of the serum samples from the K18‐hACE2 mice with SARS‐CoV‐2 antigens. (I) Schematic diagram illustrating the experimental design and sample collection for natural fomite transmission in the K18‐hACE2 mice (*n* = 6). (J) SARS‐CoV‐2 viral RNA load measured from donor's throat swab at the indicated time points by qRT‐PCR analysis of N. (K) The changes in body weight in the donors. (L) Viral load measured in the lungs and nasal turbinates at 5 dpi by qRT‐PCR analysis of N. (M) SARS‐CoV‐2 viral RNA load measured from sentinel's throat swab at the indicated time points by qRT‐PCR analysis of N. (N) The changes in body weight in the sentinel animals. (O) Reactivity of the serum samples from the sentinel animals with SARS‐CoV‐2 antigens. The limit of detection of qRT‐PCR was 0.5 copies/μl. CT, cycle threshold; dpi, days post infection.

The current standard laboratory method for testing SARS‐CoV‐2 is quantitative real‐time PCR, which measures the CT values of viral RNA as an indicator^19^, ^20^. However, there is insufficient evidence to establish a correlation between the persistence of PCR positivity and the presence of infectious SARS‐CoV‐2 particles on inanimate surfaces. To establish a laboratory fomite transmission model and test the correlation between PCR positivity and existence of infectious virus, we collected inanimate surfaces contaminated by infected animals and isolated viable virus particles. Vero cells were monitored for cytopathic effects (CPE) daily for up to 8 days, and on the 8 dpi, cell supernatants were collected for viral RNA detection (Figure 1C). Simultaneous positive results from RT‐PCR assays and CPE could confirm the presence of infectious SARS‐CoV‐2 particles. Among the 40 samples collected at 1 dpi from the four different surfaces, 9 were PCR tested positives for SARS‐CoV‐2 with CT values below 40, but infectious virus was found only from one glass and one SS sample in the CPE test. In samples collected at 2 dpi, 9 out of 40 samples tested positive for viral RNA. However, only one glass sample and one polystyrene sample exhibited a positive result in the CPE test (Figure 1D and Table S1). These results indicate that viable viruses can be retrieved from inanimate surfaces that were contaminated within the cages although at a relatively low percentage. As live virus could be detected in different fomite surfaces at different time points after the infection, we further tested the stability of the virus on different surfaces and the risk for fomite transmission.

Although there is limited evidence regarding the relationship between stability characteristics and the risk of infection via contaminated inanimate surfaces, it is generally believed that the more stable the infectious SARS‐CoV‐2 resides on various surfaces, the higher risk the fomite transmission^10^. We showed that SARS‐CoV‐2 could be detected on various contaminated inanimate surfaces (Figure 1D). Furthermore, we evaluated the stability of the SARS‐CoV‐2 Omicron variant in our experimental setup. We determined SARS‐CoV‐2 recovery yields from the inanimate surfaces of glass, SS, paper, and polystyrene at different time points. As the results indicate, no Omicron variant could be recovered from paper surfaces after a 2‐h incubation, and from any of the contaminated surfaces after 48 h. In contrast, the Omicron variant exhibited greater stability on glass and polystyrene surfaces, with viable virus titers reduced by approximately 60% within 2 h. Despite a reduction of approximately 99% in virus titer on glass and polystyrene surfaces within 24 h, the infectious virus remained at around 2 log_10_ units (Table S2). In addition, we examined the correlation between infectious virus titers and the CT values of viral RNA on various inanimate surfaces but a weak correlation was observed between infectious virus titers and CT values (Table S3). Together, these results show that the infectious virus has different stability on various inanimate surfaces and can remain infectious on the surfaces of glass, SS and polystyrene.

To investigate the risk of fomite transmission in experimental models, we placed various inanimate surfaces contaminated with the SARS‐CoV‐2 Omicron variant into the cage. Naive K18‐hACE2 mice were exposed to fomites and monitored for changes in body weight at various days postexposure (Figure 1E). It was found that fomite exposure did not result in a reduction in body weight in K18‐hACE2 mice (Figure 1F). Throat swab samples were collected from each mouse on Days 1, 3, 5, 7, and 9 post‐fomite exposure. SARS‐CoV‐2 shedding from the respiratory tract was observed in all exposure animals. Viral RNA peaked on Day 1 postexposure and remained detectable up to Day 9 postexposure (Figure 1G). Furthermore, serum samples were collected on Day 14 to detect IgG antibodies reactive to SARS‐CoV‐2 antigens. With the exception of one mouse, all mice with detectable viral loads in the swab samples also exhibited SARS‐CoV‐2 antibodies (Figure 1H). The presence of anti‐S antibodies after fomite exposure supported the notion that this route could lead to a productive infection. Overall, these findings indicate a high risk of infection in K18‐hACE2 mice exposed to virus‐contaminated fomites.

To evaluate the risk of natural fomite transmission, we introduced sentinel K18‐hACE2 mice (sentinel mice) into cages containing various inanimate surfaces that were previously exposed to infected animals (donor mice) for 5 days (Figure 1I). Virological analysis of throat swabs revealed the presence of viral RNA in the donor mice infected with the SARS‐CoV‐2 Omicron variant (Figure 1J). Weight changes were observed in all six infected donor mice, with initial weight gain slowing down after 2 dpi (Figure 1K). SARS‐CoV‐2 genomic RNA (gRNA) reached high concentrations in the lungs and nasal turbinates (Figure 1L). These data suggest that the mouse infection model of Omicron variant was well‐established, and the infected donor mice can serve as a source of virus to the inanimate surfaces. After removal of the donor mice, the sentinel mice were taken into the cage with the naturally contaminated inanimate surfaces. Throat swab samples were collected from the sentinel animals on Days 2, 4, 6, and 8 after the introduction of sentinel K18‐hACE2 mice, with three of the six sentinel animals throat swab samples testing positive for viral RNA (Figure 1M). No signs of weight loss were observed in the sentinel animals (Figure 1N). Notably, one of the six serum samples collected on Day 14 exhibited reactivity with SARS‐CoV‐2 antigens in serological analyses (Figure 1O). Taken together, our results indicate that natural transmission of SARS‐CoV‐2 can occur via fomites from the infected donor mice to sentinel healthy mice in a natural transmission setting.

This study employed the K18‐hACE2 mice model to investigate the relative contribution of fomite transmission, in a laboratory with controlled environmental conditions, whereas variations in environmental factors in natural settings may impact the transmission of SARS‐CoV‐2. In human conditions, proper hygiene measures may largely reduce the risk of fomite transmission. Due to the likelihood of SARS‐CoV‐2 transmission via fomite, mitigation efforts should prioritize proper hygienic practices, physical distancing, and monitoring of high‐risk environments (e.g., poultry, seafood, and cubed meats). Additionally, extending the resting time of goods may reduce the risk of fomite transmission. Moreover, this study underscores the importance of the K18‐hACE2 mice transmission model in assessing the transmission of novel SARS‐CoV‐2 strains amidst ongoing virus evolution. Establishing fomite exposure safety guidelines is recommended to protect public health and optimize current COVID‐19 prevention measures.

## AUTHOR CONTRIBUTIONS

**Sidi Yang**: Data curation; formal analysis; investigation; methodology; software; validation; visualization; writing—original draft. **Liu Cao**: Data curation; formal analysis; investigation; methodology; validation; visualization; writing—review and editing. **Kun Li**: Formal analysis; investigation; methodology; software; visualization. **Tiefeng Xu**: Investigation; methodology; software. **Zixiao Yang**: Methodology; software; visualization. **Yanxi Ji**: Resources; software. **Lihong Liu**: Formal analysis; investigation. **Birong Zheng**: Visualization. **Changwen Ke**: Resources. **Xiaofang Peng**: Methodology. **Hong Peng**: Data curation; validation. **Deyin Guo**: Conceptualization; project administration; resources; supervision; writing—review and editing. **Chun‐Mei Li**: Conceptualization; funding acquisition; supervision; writing—review and editing.

## ETHICS STATEMENT

Approval of animal experiments was obtained from the Institutional Animal Welfare Committee of Sun Yat‐sen University. All procedures used in animal studies complied with the guidelines and policies of the Animal Care and Use Committee of the respective research units. Work with infectious SARS‐CoV‐2 strains under BSL3 conditions was approved by the Institutional Biosafety Committee (IBC) of Sun Yat‐sen University. All sample inactivation was performed according to IBC‐approved standard operating procedures for removal of specimens from ABSL3.

## CONFLICT OF INTERESTS

The authors declare no conflict of interests.

## Supporting information

Correspondence‐Supporting Information(20250324).

## Data Availability

The data that support the findings of this study are available from the corresponding authors upon request.
