# Supplementary material for: Evidence for fomite transmission of SARS‐CoV‐2 Omicron variant in a mouse model
Source: mLife. 2025 Jun 24;4(3):332–6. doi: 10.1002/mlf2.70022 (PMC12207898; doi:10.1002/mlf2.70022)
Supplement: Supplementary file 1 — Correspondence‐Supporting Information(20250324). [file MLF2-4-332-s001.docx]

**Supporting** **Information**

**Evidence for fomite transmission of SARS-CoV-2 Omicron variant in a mouse model**

Sidi Yang^1, 2 #^, Liu Cao^2, #^, Kun Li^3^, Tiefeng Xu^2^, Zixiao Yang^2^, Yanxi Ji^2^, Lihong Liu^1^, Birong Zheng^1,3^, Changwen Ke^4^, Xiaofang Peng^4^, Hong Peng^2^, Deyin Guo^1,3,5,6 *^ & Chun-Mei Li^2^ ^*^

^1^ Guangzhou National Laboratory, Guangzhou International Bio-Island, Guangzhou 510005, China.

^2^ Centre for Infection and Immunity (CII), School of Medicine, Shenzhen Campus of Sun Yat-sen University, Shenzhen 518107, P.R. China.

^3^ Institute of Human Virology, Department of Pathogen Biology and Biosecurity, and Key Laboratory of Tropical Disease Control of Ministry of Education, Zhongshan School of Medicine, Sun Yat-sen University, Guangzhou 510080, China

^4^ Center for Disease Control and Prevention of Guangdong Province, Guangzhou 511430, P.R. China

^5^ Department of Infectious Diseases, Third Affiliated Hospital of Sun Yat-Sen University, Guangzhou 510603, P.R. China.

^6^ State Key Laboratory of Respiratory Disease, National Clinical Research Center for Respiratory Disease, Guangzhou Institute of Respiratory Health, the First Affiliated Hospital of Guangzhou Medical University, Guangzhou 510182, China.

* Correspondence to: Deyin Guo and Chun-Mei Li (guo_deyin@gzlab.ac.cn or lichm8@mail.sysu.edu.cn )

^#^ These authors contributed equally to this work.

**Running title:** Laboratory evidence of SARS-CoV-2 fomite transmission

**Materials and methods**

**Viral strains, cells and mice**

The SARS-CoV-2 Omicron variant BA.2 (GDPCC 2.00299) was isolated from a patient with COVID-19 admitted to the Eighth Affiliated Hospital of Guangzhou Medical University by Center for Disease Control and Prevention of Guangdong Province. Vero cells were obtained from American Tissue Culture Collection (ATCC) and cultured in Dulbecco’s modified eagle medium (DMEM) supplemented with 10% fetal bovine serum (FBS) at 37°C in a humidified atmosphere of 5% CO_2_. Virus propagation was performed in Vero cells in DMEM supplemented with 2% FBS. B6.Cg‐Tg (K18‐human ACE2) 2Prlmn/J (Jax strain 034860, K18‐hACE2) mice were purchased from the Jackson Laboratory. Mice were provided with sterile water and chow ad libitum and acclimatized for at least 1 week before experimental manipulation.

**Viral infections**

For the detected of contaminated viral RNA from the ambience of infected animals, K18-hACE2 mice (n=6) were intranasally (i.n.) inoculated with the Omicron BA.2 strain of SARS-CoV-2 (1 × 10^5^ TCID_50_ per mouse) and placed in a single cage containing glass, stainless steel, paper, polystyrene. Inanimate surfaces were collected for the indicated time points. Viral gRNA was measured on inanimate surfaces by qRT-PCR analysis of nucleocapsid (N).

For the isolated of viable virus from the inanimate surfaces. K18-hACE2 mice (n=6) were i.n. inoculated with the Omicron BA.2 strain of SARS-CoV-2 (1 × 10^5^ TCID_50_ per mouse) and placed in a single cage containing glass, stainless steel, paper, polystyrene. Inanimate surfaces were collected for the indicated time points. Vero cells were used to culture virus from any contaminated inanimate surfaces.

Fomite exposure was conducted by placing different inanimate surfaces that had been sprayed 5×10^5^ PFU/mouse SARS-CoV-2 (total dose per cage: 3×10^6^ PFU) into the cage. These inanimate surfaces with a dimension of 1.13 cm^2^ included stainless steel, polypropylene, glass and printing paper. Interaction of K18‐hACE2 mice (n=6) with the inanimate surfaces was monitored and confirmed within the first 5 minutes after placing it into the cage. After i.n. and fomite exposure, the animals were continuously monitored daily to record body weights, clinical symptoms and death. The throat swab samples were collected at indicated time points. All animals were euthanized 14 dpi for disease course assessment and anti-spike antibody measurement.

**Fomite transmission experiments**

Fomite transmission was examined by infecting donor K18‐hACE2 mice as described above by i.n. inoculation. Six animals per cage were housed for 5 days. At 5 dpi, donors were euthanized, and six sentinel animals were placed into the contaminated cage. K18‐hACE2 mice were followed as described above until dpi 14; bedding and cages were left undisturbed.

**Viral RNA detection**

Viral RNA was isolated from cell culture supernatants, homogenized tissue or throat swab using the Magbead Viral DNA/RNA Kit (CWBIO). SARS-CoV-2 nucleic acid detection kit (Da’an Company, Guangzhou) was used to detect the viral RNA using Applied Biosystems QuantStudio 12K Flex real-time PCR system. A sample was defined as positive for viral RNA if both N and ORF1a RT-qPCR assays gave CT values ≤ 40. The limit of detection (LOD) of this kit reached 0.5 copies/μL.

The primer sequences were N1-F: 5′-AAGAAATTCAACTCCAGGCAGC-3′; N1-R: 5′-GCTGGTTCAATCTGTCAAGCAG-3′; Prb: 5′-TCACCGCCATTGCCAGCCA-3′.

ORF1ab-F: 5′-TTAAGCGGACACAATCTTGCT-3′; ORF1ab-R: 5′-GTTGAATGTCTTCACCTTTGTTAA; Prb: 5′-CACTGTCTTCATGTTGTCGGCCCAAA

**TCID50 assay**

The TCID50 assay was determined as following (*1*), briefly, to measure the SARS-CoV-2 titres, 10-fold serial dilutions of the virus were used to inoculate Vero cell monolayers with ten replicates per dilution. The plates were incubated at 37 °C for 4 days and scored for cytopathic effect. Then TCID50 values were calculated by the Reed and Muench method (*2*).

**Virus isolation and cultures**

For virus isolation, the contaminated inanimate surfaces retrieved at desired time-points were immediately soaked with 1 mL of virus transport medium for 30 mins to elute the virus. Using 0.2 µm sterile single use filter to filter the transport medium directly into the Vero cells. Vero cells were used to culture virus from any contaminated inanimate surfaces. Vero cells were seeded in 24‐well plates (2 × 10^5^ cells/well) one day before the experiment. At indicated time points the cell supernatants were collected, and qRT-PCR used to detect SARS-CoV-2 RNA or TCID50 for determining the titration of a virus as described above.

**Enzyme-linked immunosorbent assay (ELISA)**

The concentrations of mouse Anti-S IgG antibody (Omicron) were measured using ELISA kits (Vazyme) according to the manufacturer’s instructions. Briefly, the plate was reacted for 1 h at 37℃ with mouse serum samples that has been diluted 800-fold or with positive/negative control. After washing, plates were incubated for 30 min at 37℃ with HRP-conjugated antibody. Then, plates were washed and incubated with TMB for 10 min at 37℃. The reaction was stopped by the addition of 2 M H_2_SO_4_ and the optical density at 450 nm (OD450) with noise correction at 630 nm (OD630) was immediately measured.

**The stability of SARS-CoV-2 on different surfaces**

The stability of SARS-CoV-2 on different inanimate surfaces was tested using previously described protocol (*3,4*). Briefly, a 5 μL droplet of each virus (10^8^ TCID50/ml) was applied on different surfaces with a dimension of 1.13 cm^2^. The treated surfaces were incubated at room temperature for desired time-points and were then immersed in 1 mL viral transport medium for 30 min to elute the infectious virus. The recovered virus was titrated by TCID50 assays using Vero cells as described.

**Table S1. The frequencies of viable virus recovered from inanimate surfaces.**

| Sample collection time |  | | Glass | |  | | Stainless steel | |  | | Paper | |  | | Polystyrene | |
| --- | --- | --- | --- | --- | --- | --- | --- | --- | --- | --- | --- | --- | --- | --- | --- | --- |
|  | ^#^Positive | ^*^CPE | | Virus isolation  rates (%) | ^#^Positive | CPE | | Virus isolation rates (%) | ^#^Positive | CPE | | Virus isolation rates (%) | ^#^Positive | CPE | | Virus isolation rates (%) |
| Day 1 | 2/10 | 1/10 | | 10% | 1/10 | 1/10 | | 10% | 5/10 | 0/10 | | - | 1/10 | 0/10 | | - |
| Day 2 | 3/10 | 1/10 | | 10% | 0/10 | 0/10 | | - | 3/10 | 0/10 | | - | 3/10 | 1/10 | | 10% |

All the virus isolation using Vero cells. After 8 days the cell supernatants were collected, and RT-qPCR used to detect SARS-CoV-2 RNA.

^#^ Viral RNA positive/total number of samples.

^*^ CPE positive/total number of samples.

**Table S2. Stability of SARS-CoV-2 Omicron variant** **on different inanimate surfaces.**

| Time | Virus titer (Log TCID_50_/mL) ^#^ | | | | | | | | | | | |
| --- | --- | --- | --- | --- | --- | --- | --- | --- | --- | --- | --- | --- |
|  | Glass | | | Stainless steel | | | Paper | | | Polystyrene | | |
|  | Mean | ±SD | % Reduction in viral titre | Mean | ±SD | % Reduction in viral titre | Mean | ±SD | % Reduction in viral titre | Mean | ±SD | % Reduction in viral titre |
| 0 h | 5.319 | 0.060 | NA | 5.308 | 0.064 | NA | 5.300 | 0.075 | NA | 5.353 | 0.042 | NA |
| 2 h | 4.833 | 0.095 | 66.91% | 4.240 | 0.058 | 91.46% | ^*^0.766 | - | 99.97% | 4.836 | 0.326 | 61.32% |
| 24 h | 2.932 | 0.403 | 99.36% | 2.432 | 0.125 | 99.86% | U | - | ＞99.99% | 3.066 | 0.262 | 99.40% |
| 48 h | U | - | ＞99.99% | U | - | ＞99.99% | U | - | ＞99.99% | U | - | ＞99.99% |

^#^ All the virus titers were titrated using Vero cells. All experimental studies were done in three independent triplicates. Detection limit of a typical TCID50 assay is 100 TCID50/mL. NA: Not Available, U: undetectable.

^*^ Only one of the triplicate reactions was positive in the TCID50 assay.

**Table S3. Comparison of detection of the Real-Time SARS-CoV-2 quantitative and virus titer on different inanimate surfaces.**

| Time | Glass | | | | Stainless steel | | | | Paper | | | | Polystyrene | | | |
| --- | --- | --- | --- | --- | --- | --- | --- | --- | --- | --- | --- | --- | --- | --- | --- | --- |
|  | CT value | | Virus titer (Log TCID_50_/mL) ^#^ | | CT value | | Virus titer (Log TCID_50_/mL) ^#^ | | CT value | | Virus titer (Log TCID_50_/mL) ^#^ | | CT value | | Virus titer (Log TCID_50_/mL) ^#^ | |
|  | Mean | ±SD | Mean | ±SD | Mean | ±SD | Mean | ±SD | Mean | ±SD | Mean | ±SD | Mean | ±SD | Mean | ±SD |
| 0 h | 22.697 | 0.044 | 5.319 | 0.060 | 24.167 | 0.306 | 5.308 | 0.064 | 30.438 | 0.672 | 5.300 | 0.075 | 23.441 | 0.486 | 5.353 | 0.042 |
| 2 h | 23.404 | 0.565 | 4.833 | 0.095 | 24.495 | 0.251 | 4.240 | 0.058 | 34.882 | 0.462 | ^*^0.766 | - | 24.758 | 0.335 | 4.836 | 0.326 |
| 24 h | 23.596 | 0.432 | 2.932 | 0.403 | 23.704 | 0.183 | 2.432 | 0.125 | 34.428 | 0.968 | U | - | 24.125 | 0.492 | 3.066 | 0.262 |
| 48 h | 24.251 | 1.965 | U | - | 22.944 | 0.397 | U | - | 31.705 | 1.161 | U | - | 20.974 | 0.922 | U | - |

All experimental studies were done in three independent triplicates. CT, cycle threshold values for viral RNA.

^#^ Detection limit of a typical TCID50 assay is 100 TCID50/mL. U: undetectable.

^*^ Only one of the triplicate reactions was positive in the TCID50 assay.

**References**

1. S. Gong *et al.*, Human-Derived A/Guangdong/Th005/2017 (H7N9) Exhibits Extremely High Replication in the Lungs of Ferrets and Is Highly Pathogenic in Chickens. *Viruses* **11**, (2019).

2. S. Biacchesi *et al.*, Rapid human metapneumovirus microneutralization assay based on green fluorescent protein expression. *J Virol Methods* **128**, 192-197 (2005).

3. S. Behzadinasab, A. Chin, M. Hosseini, L. Poon, W. A. Ducker, A Surface Coating that Rapidly Inactivates SARS-CoV-2. *ACS Appl Mater Interfaces* **12**, 34723-34727 (2020).

4. A. W. H. Chin, A. M. Y. Lai, M. Peiris, L. L. Man Poon, Increased Stability of SARS-CoV-2 Omicron Variant over Ancestral Strain. *Emerg Infect Dis* **28**, 1515-1517 (2022).
